# Fecal neuraminidase correlates with inflammatory signatures and microbiome shifts in preclinical Crohn’s disease

**DOI:** 10.1080/19490976.2026.2755166

**Published:** 2026-11-05

**Authors:** Kaiwen Mu, Cong Phi Dang, Mingyue Xue, Qilong Li, Hien Q. Huynh, Anne M. Griffiths, Dan Turner, Paul Moayyedi, A. Hillary Steinhart, Levinus A. Dieleman, Colette Deslandres, Alain Bitton, David R. Mack, Kevan Jacobson, Maria Cino, Guy Aumais, Remo Panaccione, Brian Bressler, Sun-Ho Lee, Kenneth Croitoru, Williams Turpin

**Affiliations:** a Division of Gastroenterology & Hepatology, Temerty Faculty of Medicine, University of Toronto, Toronto, Ontario, Canada; b Zane Cohen Centre for Digestive Diseases, Mount Sinai Hospital, Toronto, Ontario, Canada; c Division of Gastroenterology and Nutrition, Department of Pediatrics, Faculty of Medicine and Dentistry, University of Alberta, Edmonton, Alberta, Canada; d IBD Centre, The Hospital for Sick Children, Department of Pediatrics, Faculty of Medicine, University of Toronto, Toronto, Ontario, Canada; e The Juliet Keidan Institute of Pediatric Gastroenterology and Nutrition, The Eisenberg R&D Authority, Shaare Zedek Medical Center, The Hebrew University of Jerusalem, Jerusalem, Israel; f Department of Medicine, Farncombe Family Digestive Health Research Institute, McMaster University, Hamilton, Ontario, Canada; g Division of Gastroenterology, Department of Medicine, University of Alberta, Edmonton, Alberta, Canada; h Division of Gastroenterology, Hepatology and Nutrition, Department of Pediatrics, CHU Sainte-Justine, University of Montreal, Montreal, Quebec, Canada; i Division of Gastroenterology and Hepatology, McGill University Health Centre, Montreal, Quebec, Canada; j Division of Gastroenterology, Hepatology & Nutrition, Children’s Hospital of Eastern Ontario and University of Ottawa, Ottawa, Ontario, Canada; k Division of Gastroenterology, Department of Medicine, University of British Columbia, BC Children’s Hospital, and BC Children’s Hospital Research Institute, Vancouver, British Columbia, Canada; l Department of Medicine, Hôpital Maisonneuve-Rosemont, University of Montreal, Montreal, Quebec, Canada; m Division of Gastroenterology, Department of Medicine, University of Calgary, Calgary, Alberta, Canada; n University of British Columbia, Vancouver, British Columbia, Canada; o Department of Nutritional Sciences, Temerty Faculty of Medicine, University of Toronto, Toronto, Ontario, Canada

**Keywords:** Crohn’s disease, neuraminidase, mucosal inflammation, host-microbiome interactions, GEM cohort

## Abstract

The mucus layer, rich in mucin glycoproteins, serves as a critical barrier to microbial invasion. Neuraminidases cleave terminal sialic acids from mucin glycans, a process that may influence mucus integrity, epithelial exposure to microbial antigens, and mucosal immune responses, making fecal neuraminidase activity a biologically relevant functional readout of host-microbe interactions in the context of Crohn’s disease (CD) pathogenesis. We evaluated whether fecal neuraminidase activity is associated with future CD risk and examine its relationships with inflammatory and microbial signatures relevant to early disease development. Within the prospective GEM cohort, 69 individuals who later developed CD were matched to 69 healthy first-degree relative controls. Fecal neuraminidase activity was measured using a fluorometric assay. Conditional logistic regression assessed its association with future CD risk. Partial Spearman correlations evaluated relationships with 446 serum proteins (OLINK), followed by pathway enrichment analysis, and with fecal calprotectin (FCP) and gut microbial composition (16S rRNA sequencing). Fecal neuraminidase activity was positively associated with future CD (OR = 1.541; 95% CI: 1.05–2.25; *p* = 0.026). Proteins correlated with neuraminidase activity were enriched in immune and inflammatory pathways. Neuraminidase activity was positively correlated positively with FCP (*p* ≤ 0.05), and its association with future CD was attenuated after adjustment for FCP. Fourteen microbial taxa were significantly correlated with neuraminidase activity (*q* ≤ 0.05). Fecal neuraminidase activity's relationships with inflammatory markers and microbial taxa support its potential relevance as a functional marker of host-microbe alterations during preclinical CD.

## Graphical abstract

**Figure:**
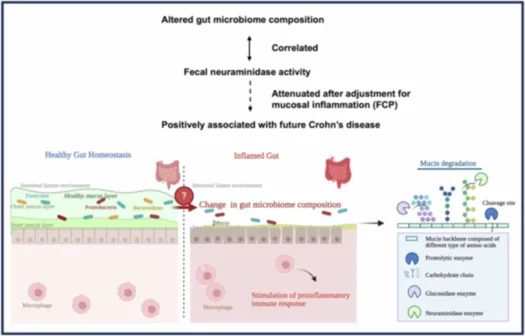

## 1. Introduction

Crohn’s disease (CD) is a chronic inflammatory bowel disease (IBD) characterized by inflammation of the gastrointestinal tract, leading to a range of symptoms such as abdominal pain, diarrhea, and weight loss.^1^ Despite extensive research, the precise mechanisms underlying CD pathogenesis remain unclear. It is recognized that alterations in the gut microbiome, intestinal permeability, and immune responses play pivotal roles in disease development.^2-4^ One key aspect of gut physiology that has recently gained focus is the intestinal mucus layer, which serves as a protective barrier against pathogens, toxins, and harmful microorganisms.^5-7^ The degradation of this protective mucus layer, particularly through the action of enzymes like neuraminidases, has been proposed as a potential contributor to CD pathogenesis.^8-10^

Neuraminidase (sialidase) is an enzyme that cleaves sialic acid residues from glycoproteins and glycolipids on the cell surface, including mucins of the intestinal mucus layer. It is primarily derived from gut microbial activity, although a minor contribution from host-derived sialidases released during epithelial turnover cannot be excluded.^11-13^ In addition to its role in mucosal damage, neuraminidase activity has been associated with inflammatory responses in both human and animal models.^11–13^ When sialic acid residues are cleaved from cell surface glycoproteins, this may expose underlying molecules that act as damage-associated molecular patterns (DAMPs).^14-16^ These DAMPs are recognized by immune receptors, triggering innate immune system activation and the release of pro-inflammatory cytokines, thereby contributing to the chronic inflammatory state characteristic of CD.^17-19^

Recent studies suggest that an imbalance in the gut microbiota may influence neuraminidase activity, with certain microbial species potentially contributing to the observed activity.^20-24^ Since neuraminidase activity has been implicated in mucosal damage and immune activation, measuring fecal neuraminidase activity could provide valuable insights into the early changes associated with CD risk. These findings suggest that fecal neuraminidase activity may represent a functional feature associated with future CD and provide biological insight into host-microbe interactions during the preclinical phase of disease.

In this pilot study, we utilized stool samples from the Genetic, Environment, and Microbial (GEM) cohort to compare fecal neuraminidase activity between individuals who subsequently developed CD (pre-CD) and healthy matched controls. We further evaluated its association with future CD development and its correlation with established GEM biomarkers to better understand the underlying biological mechanisms. To further characterize the microbial context of fecal neuraminidase activity, we examined its correlation with gut microbiota composition.

## 2. Materials and methodology

### 2.1. Study design

The GEM Project is a large prospective cohort designed to investigate genetic, environmental, and microbial factors associated with CD development.^25^ ^,^ ^26^ This study included a nested case-control subset of first-degree relatives (FDRs) recruited between 2008 and 2020, in which individuals who later developed CD (pre-CD, *n* = 69) were matched to FDRs who remained healthy (*n* = 69) based on age, sex, geographic location, and follow-up duration. All participants were free of gastrointestinal disease at recruitment.^27^ Written informed consent was obtained from all participants, and the study was approved by the Mount Sinai Hospital Research Ethics Board and participating centers. Additional cohort details, recruitment criteria, and follow-up procedures are provided in the Supplementary Methods.

### 2.2. Fecal neuraminidase activity assessment

Fecal samples (~100 mg) were homogenized in pre-reduced phosphate-buffered saline under anaerobic conditions and stored at −80 °C until analysis. Fecal neuraminidase activity was measured using a fluorometric neuraminidase assay kit (Abcam, MA, USA; catalog number ab138888) according to the manufacturer’s instructions. Activity was quantified using a standard curve and measured in triplicate. Total protein concentration was determined using the Bradford assay, and neuraminidase activity was normalized to protein content (mU/mg protein) to account for differences in sample input and extraction efficiency, consistent with previously published protocols.^28-30^ One pre-CD sample failed protein quantification and was excluded, resulting in 68 pre-CD samples. Additional methodological details are provided in the Supplementary Methods.

### 2.3. Serum protein quantification

Proteomic analysis was performed on baseline serum samples using the proximity extension assay (PEA) technology in combination with quantitative real-time PCR (Olink® Proteomics, Uppsala, Sweden) (*n* = 92). In total, 446 protein markers were quantified across five Olink® Target 96 panels, as described in prior work.^31^ ^,^ ^32^ Additional details are provided in the Supplementary Materials.

### 2.4. Pathway enrichment analysis

To assess the biological relevance of these associations, pathway enrichment analysis was carried out using the Appyters web platform (https://appyters.maayanlab.cloud/#/). The analysis focused on proteins that exhibited correlations meeting the exploratory threshold with the fecal neuraminidase activity (*p* < 0.1). Pathway mapping was performed against the WikiPathways_2019_Human gene set library.^33-35^

### 2.5. Assessment of FCP

FCP levels were measured using a commercial enzyme-linked immunosorbent assay (ELIZA) kit (BÜHLMANN fCAL, Schönbuch, Switzerland), following the manufacturer’s instructions.^27^ ^,^ ^32^ Additional details are provided in the Supplementary Materials (*n* = 136).

### 2.6. Assessment of intestinal barrier (LMR)

Intestinal barrier function was assessed using the lactulose to mannitol ratio (LMR), following previous protocols.^32^ ^,^ ^36-38^ Participants consumed a solution containing lactulose, mannitol, sucrose, and flavored drink crystals before bed, and urine samples were collected the next morning (*n* = 127). Lactulose and mannitol concentrations in urine were analyzed using high-pressure liquid chromatography.^37^ ^,^ ^39^ The LMR was calculated as the fractional excretion of lactulose divided by that of mannitol.^36^

### 2.7. Taxonomic profiling of the gut microbiota

Fecal DNA was extracted using the QIAamp DNA Stool Mini Kit (Qiagen, Germany). The V4 region of the bacterial 16S rRNA gene was amplified using the 515F/806R primers and sequenced (2 × 150 bp) on the Illumina MiSeq platform.^25^ ^,^ ^40^ Sequence processing was performed using QIIME2, with DADA2 for denoising, and taxonomic classification was assigned using the SILVA 132 database. Quality filtering and downstream microbiome analyzes were performed using previously described approaches. Additional details regarding sequence processing, filtering criteria, and statistical handling of microbiome data are provided in the Supplementary Methods (*n* = 135).

### 2.8. Statistical analysis

Conditional logistic regression was used to examine the association between fecal neuraminidase activity and the risk of developing CD, accounting for the matched nested-group design. Neuraminidase activity was standardized using z-score normalization prior to analysis to allow effect estimates to be expressed per standard deviation increase. Given the established association between FCP and intestinal inflammation, additional conditional logistic regression analyzes were performed to evaluate the relationships of neuraminidase activity and FCP with future CD. Models included neuraminidase activity and FCP individually, followed by a mutually adjusted model including both variables to assess whether their associations with future CD were independent of one another. FCP was also standardized using z-score normalization to facilitate comparison of effect estimates.

Partial Spearman correlation analysis was conducted to assess the association between 446 serum proteins and neuraminidase activity while accounting for the nested group, followed by pathway enrichment analysis to identify key biological pathways involved. We further explored the relationship between neuraminidase activity and CD risk biomarkers related to these pathways, including gut permeability (LMR) andFCP levels. Finally, partial Spearman correlation analysis was employed to investigate correlations between fecal neuraminidase activity and gut microbiota composition, as determined by fecal 16S rRNA sequencing. *P*-values were adjusted for multiple comparisons using the Benjamini–Hochberg false discovery rate (FDR) method, with *q* < 0.05 considered statistically significant.

## 3. Results

### 3.1. Baseline characteristics of the nested case–control cohort

The study cohort described here is a subset of the GEM Project cohort, which included 69 individuals who subsequently developed CD and 69 matched FDRs who remained healthy. The median age at recruitment was 15.0 (IQR 10.0–22.0), with 56.5% identifying as female. Additionally, 84.8% were recruited in Canada, 11.6% in Israel, 2.2% in the USA, and 1.4% in Ireland. The median follow-up was over 3 years for both the healthy matched control (HMC) group and the pre-CD group (Table 1).

**Table 1.** Demographics of nested cohort used for this study.

Variable		Remained Healthy FDR (*N* = 69)	pre-CD FDR (*N* = 69)
Age at recruitment	Median (IQR)^a^	15 [10.0–22.0]	15 [10.0–21.0]
Sex, female^†^	*n* (%)	39 (56.5%)	39 (56.5%)
Recruitment country^†^			
	Canada	60 (87.0%)	57 (82.6%)
	Ireland	1 (1.4%)	1 (1.4%)
	USA	–	3 (4.3%)
	Israel	8 (11.6%)	8 (11.6%)
Follow-up duration (years)	Median (IQR)^a^	3.22 [1.64–5.04]	3.28 [1.70–5.51]

Categorical variables are presented as numbers and percentages; continuous variables are presented as median and interquartile range.

CD, Crohn’s disease; FDRs, first-degree relatives.

^a^ The data is presented as the median, followed by the interquartile range (IQR) in square brackets.

^†^ The data is presented as the number of cases, followed by the percentage of each group in parentheses.

### 3.2. Fecal neuraminidase activity is positively associated with future CD

The range of fecal neuraminidase activity levels in health matched control was between 14.92 and 231.28 mU/mg of protein, while in pre-CD, it ranged from 17.64 to 298.75 mU/mg of protein in human stool samples. Direct paired comparison showed that fecal neuraminidase activity was significantly higher in the pre-CD group than in matched healthy controls (median 98.07 mU/mg [IQR 67.00–133.87] vs 79.19 mU/mg [58.22–104.80]; Wilcoxon signed-rank test, *p* = 0.027). Conditional logistic regression was utilized to assess the association between fecal neuraminidase activity levels and future CD onset. The results indicated that increased fecal neuraminidase activity levels at recruitment were significantly associated with an increased risk of developing CD (Odds ratio = 1.541; 95% CI: 1.05–2.25; *p* = 0.026) (Figure 1).

**Figure 1.**
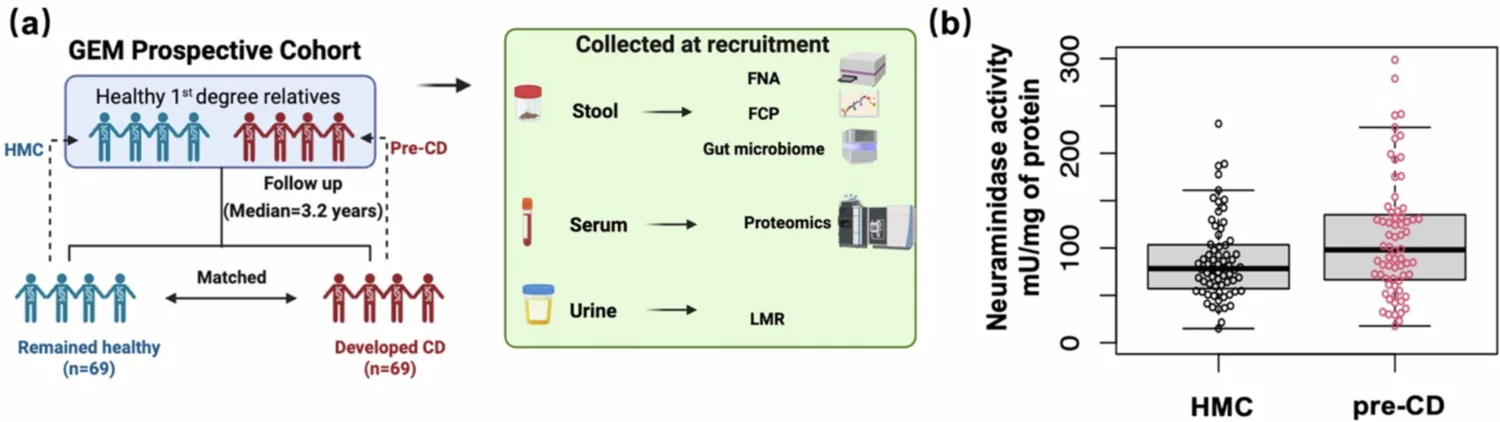
(a) Human serum, stool and urine samples were collected at the time of recruitment as part of the GEM project from individuals who later developed CD (the pre-CD group) and individuals who remained healthy (the HMC group). The scheme was created using Biorender.com. (b) Comparison of fecal neuraminidase activity in healthy matched control stool (HMC in black) (*n* = 69) and subjects that developed CD (pre-CD in red) stool samples (*n* = 68). Conditional logistic regression was utilized to assess the association between fecal neuraminidase activity and future Crohn’s disease (CD). For regression analyzes, fecal neuraminidase activity was standardized using Z-score standardization. Non-transformed values are shown in figures. The Y-axis represents the neuraminidase activity, expressed as mU/mg of protein in human stool samples. The interquartile range (IQR), represented by the box, spans from Q1 to Q3 and encompasses the median of the data. Each dots represent the mean fecal neuraminidase activity of a given participant. Abbreviations: GEM: genetic, environmental and microbial; HMC: healthy matched control; CD: Crohn’s disease; IQR: interquartile range; Q: quartile; LMR: lactulose/mannitol ratio.

Subgroup analysis was performed based on the median time to diagnosis (3.28 years) to evaluate whether the association between fecal neuraminidase activity and Crohn’s disease risk differs by the temporal proximity to diagnosis, and to explore whether elevated fecal neuraminidase activity may represent an early event in disease development. Subgroup analysis was performed by stratifying participants into two groups: those diagnosed earlier (*n* = 34 pairs) and those diagnosed later (*n* = 34 pairs) (**Supplementary Figure 1**). In the subgroup with a diagnosis date of less than 3.28 years after recruitment, the results showed a trend to significance (Odds ratio = 1.791; 95% CI: 0.991–3.238; two-sided test *p* = 0.054), indicating an association between increased neuraminidase levels and an elevated risk of CD. In the subgroup with a diagnosis date greater than 3.28 years after recruitment, the direction of effect remained consistent, though not significant (Odds ratio = 1.218; 95% CI: 0.752–1.973; two-sided test *p* = 0.424). Fecal neuraminidase activity was not significantly correlated with freezer storage duration, supporting the stability of fecal neuraminidase activity in frozen samples (**Supplementary Figure 2**).

### 3.3. A positive correlation between fecal neuraminidase activity and mucosal inflammation was identified through serum proteomic and fecal calprotectin assessments

The partial Spearman correlation between 446 serum proteins and fecal neuraminidase activity identified a total of 7 serum proteins with significant positive correlations with fecal neuraminidase activity, while 14 serum proteins showed significant negative correlations with fecal neuraminidase activity (*p* ≤ 0.05) (**Supplementary Table 1**) (sample size *n* = 92). To gain biological insight and understand the correlation between fecal neuraminidase activity and the host proteomic profile, we performed a pathway enrichment analysis of serum proteins positively (Figure 2(a)) and negatively (Figure 2(b)) correlated with fecal neuraminidase activity, respectively. Using the WikiPathways (WP) Human 2019 database, several immune and inflammation related pathways were found to be significantly enriched. The top 10 enriched terms for the input gene set are displayed based on the -log10(*p*-value) (FDR-adjusted *p* ≤ 0.05).

**Figure 2.**
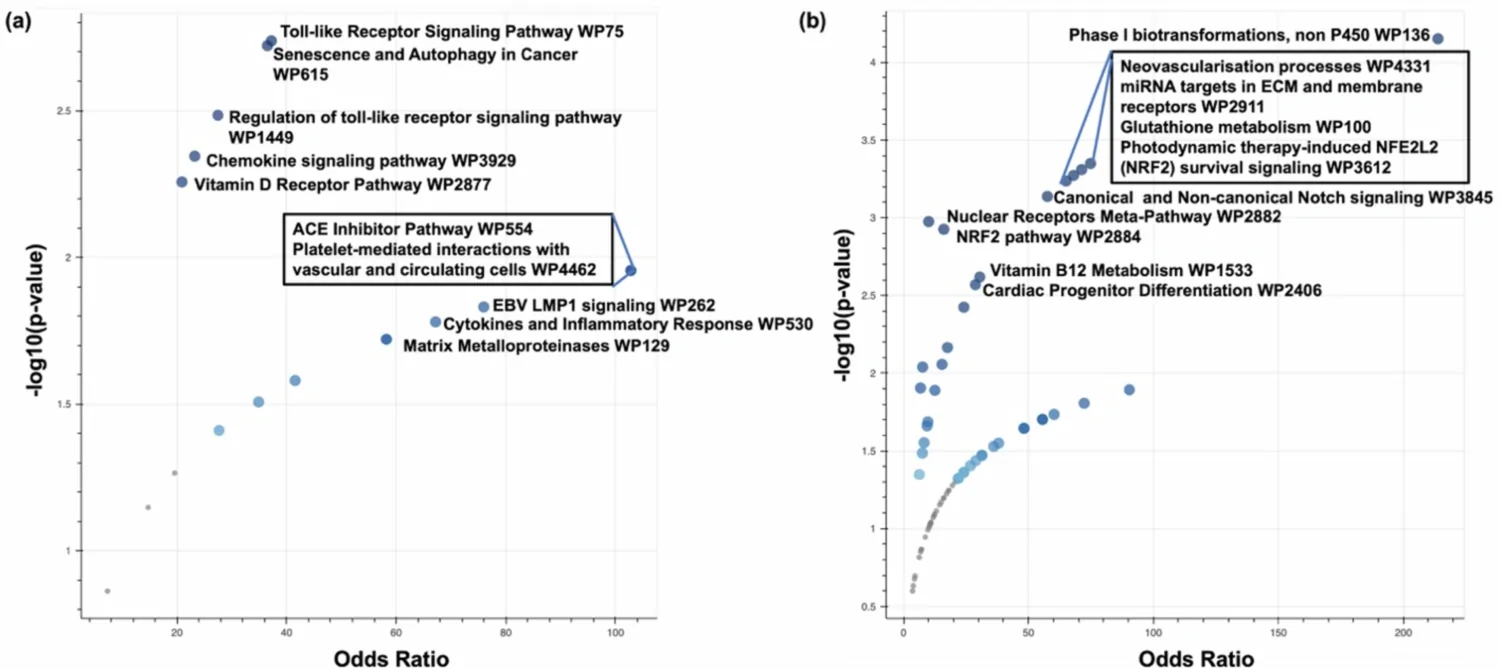
Volcano plot of pathway enrichment results based on the WikiPathways_2019_Human gene set library. (a) depicts the enriched pathways derived from the 14 serum proteins that exhibited positive correlations with fecal neuraminidase activity (*p* ≤ 0.1). (b) depicts the enriched pathways derived from the 30 serum proteins that exhibited negative correlations with fecal neuraminidase activity (*p* ≤ 0.1). Each point represents a pathway term, with its odds ratio plotted on the x-axis and –log_10_(*p*-value) on the y-axis. The top 10 enriched terms for the input gene set are displayed based on the -log_10_(*p*-value). Larger and darker points indicate greater enrichment significance.

Proteomic pathway analysis demonstrated distinct host processes connected to fecal neuraminidase activity. Proteins negatively correlated with fecal neuraminidase activity were enriched in pathways connected to antioxidant defense and cellular regulation, including glutathione metabolism (WP100), NRF2 signaling (WP2884), nuclear receptor signaling (WP2882), and canonical and non-canonical Notch signaling (WP3845), suggesting that lower fecal neuraminidase activity is correlated with enhanced redox balance and immune regulatory processes. In contrast, proteins positively correlated with fecal neuraminidase activity were enriched in immune and inflammatory pathways such as toll-like receptor signaling (WP75, WP1449), chemokine signaling (WP3929), cytokines and inflammatory response (WP530), and platelet-mediated interactions with immune and vascular cells (WP4462), indicating that elevated fecal neuraminidase activity is correlated with proteins involved in innate immune responses and leukocyte recruitment.

A significant positive correlation was observed between fecal neuraminidase activity and FCP, with a partial Spearman correlation coefficient equaling 0.29 (*p* = 0.0004, sample size *n* = 136) (Figure 3). The correlation between fecal neuraminidase activity and FCP in the pre-CD and HMC groups is shown separately in **Supplementary Figure 3**.

**Figure 3.**
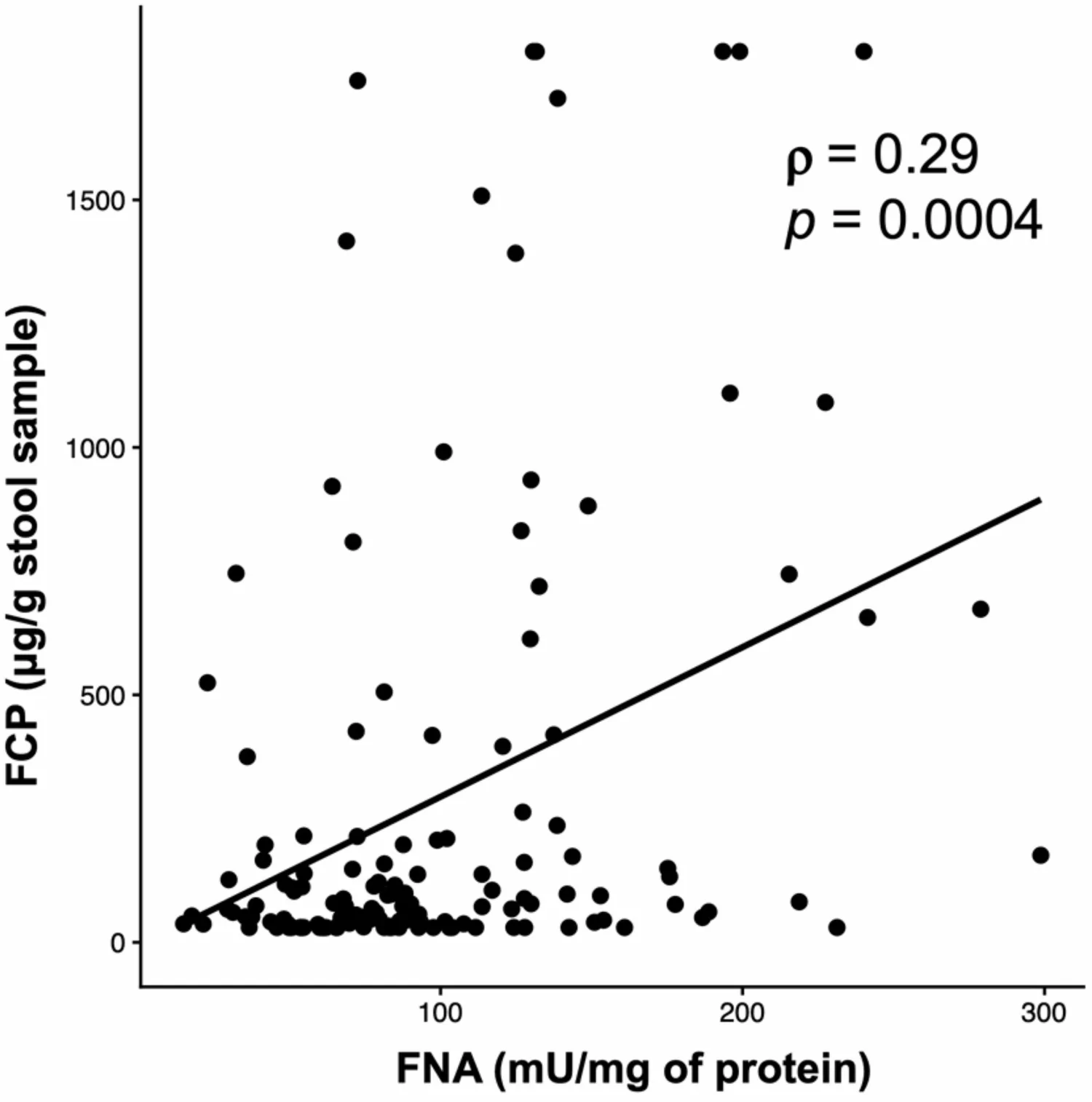
Scatter plot illustrating the relationship between fecal neuraminidase activity (FNA) in human stool samples (x-axis) and fecal calprotectin (FCP) (y-axis). Partial Spearman correlation analysis revealed a significant positive correlation between fecal neuraminidase activity and FCP (*ρ* = 0.29, *p* = 0.0004, *n* = 136). Each point represents an individual sample, with a line included to illustrate the trend. Abbreviations: FNA: fecal neuraminidase activity; FCP: fecal calprotectin.

### 3.4. Relationship between fecal neuraminidase activity, FCP, and future CD

Given the observed relationship between fecal neuraminidase activity and inflammatory markers, we further examined the association between fecal neuraminidase activity, FCP, and future CD. In conditional logistic regression, higher fecal neuraminidase activity was associated with an increased risk of future CD (OR = 1.54, 95% CI 1.05–2.25; *p* = 0.026). FCP was also associated with future CD when examined separately (OR = 2.66, 95% CI 1.42–4.98; *p* = 0.002).

When fecal neuraminidase activity and FCP were simultaneously included in the model, the association between fecal neuraminidase activity and future CD was attenuated (OR = 1.07, 95% CI 0.70–1.61; *p* = 0.760), whereas the association between FCP and future CD remained significant (OR = 2.55, 95% CI 1.30–5.00; *p* = 0.006) (Figure 4). Thus, the association between fecal neuraminidase activity and future CD was not retained after accounting for FCP. As fecal neuraminidase activity and FCP were measured concurrently at baseline, these analyzes do not establish the nature or direction of the relationship between these measures.

**Figure 4.**
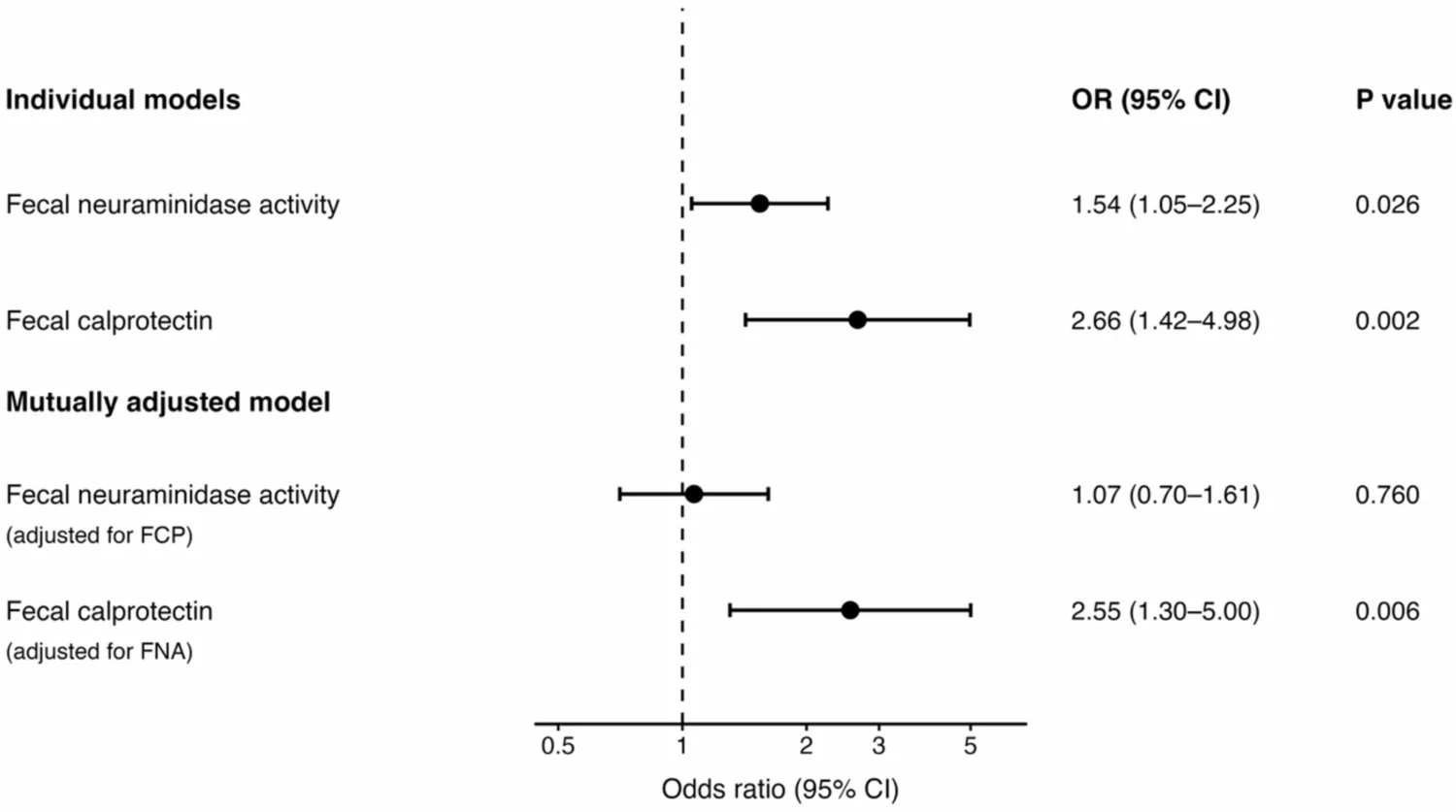
Forest plot showing the associations of fecal neuraminidase activity and fecal calprotectin with future Crohn’s disease, presented as odds ratios (ORs) and 95% confidence intervals (CIs) derived from conditional logistic regression accounting for the matched nested-group design. Fecal neuraminidase activity (FNA) and fecal calprotectin (FCP) were examined individually and in a mutually adjusted model (*n* = 136). In the mutually adjusted model, the FNA estimate was adjusted for FCP, and the FCP estimate was adjusted for FNA. Solid circles indicate ORs, horizontal lines indicate 95% CIs, and the dashed vertical line indicates the null value (OR = 1). Abbreviations: FNA: fecal neuraminidase activity; FCP: fecal calprotectin; CI: confidence intervals.

### 3.5. Fecal neuraminidase activity correlates with the relative abundance of several bacterial taxa

Positive correlations with higher neuraminidase activity were observed for several taxa, including *Oscillospiraceae Flavonifractor*, *Lachnospiraceae Ruminococcus gnavus* group, and *Lachnospiraceae Lachnoclostridium* (Figure 5). Detailed results are provided in **Supplementary Table 2.** In contrast, multiple members of the *Oscillospiraceae*, *Ruminococcaceae*, and *Clostridia* groups were negatively correlated with increasing neuraminidase activity. Partial Spearman correlation analyzes demonstrated that these correlations remained significant after accounting for intestinal inflammation (FCP). Collectively, these findings reveal distinct microbial compositional signatures associated with fecal neuraminidase activity, suggesting a potential functional relationship between microbial community structure and enzymatic activity in the gut.

**Figure 5.**
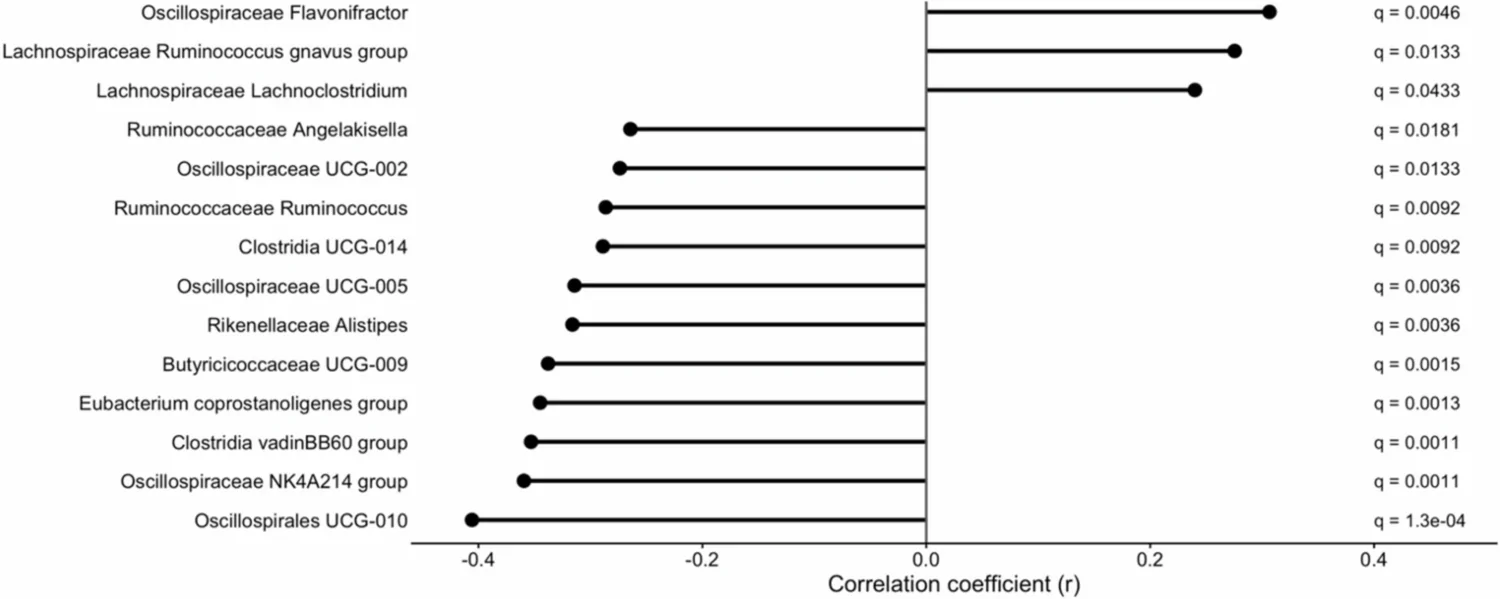
Partial correlations between fecal neuraminidase activity and the relative abundance of gut bacterial taxa. Each line represents the correlation for a bacterial taxon, with circles denoting the correlation coefficient. All correlations shown were statistically significant (*q* < 0.05).

## 4. Discussion

This study identifies fecal neuraminidase activity as a functional feature associated with an increased risk of developing CD prior to clinical diagnosis. To investigate potential biological mechanisms underlying this association, we performed exploratory proteomic and microbiome analyzes. Among the exploratory microbiome findings, the positive correlation with *Ruminococcus gnavus*, a well-characterized mucin-degrading bacterium, represents a biologically plausible link connecting microbial enzymatic activity, mucus barrier alteration, and intestinal inflammation.^41-43^

*R. gnavus* is frequently enriched in inflammatory bowel disease and is known to encode an intramolecular trans-sialidase (NanH) that cleaves terminal sialic acids from mucin glycans, facilitating mucin utilization as a nutrient source.^44^ In the present study, the relative abundance of the *R. gnavus* group was positively correlated with fecal neuraminidase activity, consistent with its established role in mucus erosion.^45-47^ Experimental studies have demonstrated that removal of sialic acid residues renders mucins more susceptible to degradation by additional bacterial glycosidases and proteases, thereby weakening the protective mucus layer.^24^ ^,^ ^48-50^ Together with previous experimental studies, these findings are consistent with the hypothesis that increased neuraminidase activity may be associated with enhanced mucin degradation during the preclinical phase of CD; however, mucin desialylation and mucus integrity were not directly assessed in the present study.

Loss or thinning of the mucus barrier may permit bacteria to reside in closer proximity to the intestinal epithelium, thereby increasing host exposure to microbial- and damage-associated molecular patterns.^13^ ^,^ ^51^ Sialic acids on mucosal glycoproteins play a critical role in immune regulation by limiting excessive immune activation and promoting immune tolerance. Prior experimental studies suggest that desialylation of these structures by microbial neuraminidases may therefore remove an important immunoregulatory signal, facilitating engagement of epithelial and immune pattern recognition receptors and amplifying inflammatory signaling.^13^ ^,^ ^51^ However, these mechanisms were not directly examined in our study.

Consistent with this interpretation, fecal neuraminidase activity was correlated with proteomic signatures enriched in innate immune and inflammatory pathways, including Toll-like receptor, chemokine, cytokine, and Epstein Barr virus-related signaling.^52^ ^,^ ^53^ These pathways are well recognized contributors to CD pathogenesis and are consistent with immune related biological processes rather than direct epithelial injury. Supporting this interpretation, neuraminidase activity was positively correlated with FCP, a robust marker of neutrophil-driven mucosal inflammation.^8-10^ In conditional logistic regression, the association between fecal neuraminidase activity and future CD was attenuated after accounting for FCP, whereas FCP remained associated with future CD after accounting for fecal neuraminidase activity. These findings provide additional context for interpreting increased fecal neuraminidase activity during the preclinical phase of CD. Rather than establishing neuraminidase activity as a marker independent of intestinal inflammation, the findings suggest that increased neuraminidase activity occurs within a broader preclinical state characterized by emerging intestinal inflammatory activity. Together with the observed inflammatory proteomic signatures, these results support a relationship between functional alterations and the evolving preclinical intestinal environment. Because fecal neuraminidase activity and FCP were measured concurrently at baseline, their temporal and mechanistic relationship cannot be determined from the present study.

Notably, fecal neuraminidase activity was not significantly associated with marker of gut barrier integrity (**Supplementary figure 4**), suggesting that its association with CD risk may relate more closely to immune-related processes than to overt epithelial damage. This interpretation is consistent with mechanistic studies demonstrating that neuraminidase-driven desialylation can reduce intestinal alkaline phosphatase activity, amplify TLR4-dependent inflammatory signaling, and promote colitis even in the absence of clear evidence of barrier disruption.^8-10^ These findings are consistent with a model in which microbial neuraminidase activity may contribute to, or arise within, a pro-inflammatory mucosal environment through alterations in mucus composition and immune sensing at the epithelial interface.

Taken together, these results identify elevated fecal neuraminidase activity as a functional enzymatic feature associated with the preclinical phase of CD and correlated with mucin-degrading bacteria such as *R. gnavus*, inflammatory proteomic signatures, and mucosal inflammation. The integrated microbiome, proteomic, and inflammatory findings support fecal neuraminidase activity as a biologically informative readout of host–microbe interactions during the evolving preclinical intestinal environment and highlight the potential value of functional microbiome measurements alongside taxonomic profiling for characterizing early changes associated with CD development.

This study is limited by its relatively small sample size and by the focus on a single mucin glycan-degrading enzyme, which may not fully capture the broader spectrum of microbial mucin degradation pathways relevant to CD. Future studies should investigate additional glycan-degrading enzymes. In our current study, fecal neuraminidase activity was measured as a total functional enzymatic readout. Because fecal neuraminidase activity reflects an aggregate enzymatic signal that may derive from both microbial sources and host-derived sialidases, it should be interpreted as a functional biomarker of host-microbe interactions rather than a strictly microbe-specific marker. Future studies should also investigate how dietary factors may influence fecal neuraminidase activity, as diet could represent a potential modifiable factor affecting intestinal enzymatic activity. Finally, despite the prospective design demonstrating that changes in fecal neuraminidase activity precede clinical diagnosis, this observational study cannot establish a causal role in disease development. An additional limitation is the absence of an independent cohort for external replication. Validation of the prediagnostic association will require independent prospective cohorts with stool samples collected before CD onset. Such studies will be important for establishing the generalizability, reproducibility, and potential clinical relevance of these findings.

## 5. Conclusion

In conclusion, our results suggest that fecal neuraminidase activity is associated with increased CD risk and may reflect mucosal inflammation, as indicated by its relationship with FCP. These findings highlight the potential value of fecal enzymatic activity as a functional readout of host-microbe interactions and provide insights into biological alterations occurring during the preclinical phase of CD.

## Supplementary Material

Supplementary Material Supplementary Material

20260821_Resubmission_Supporting_material_FNA_paper__WTnotrackchanges.docx 20260821_Resubmission_Supporting_material_FNA_paper__WTnotrackchanges.docx

## Data Availability

Requests for access to raw or analyzed data must comply with the guidelines outlined at http://www.gemproject.ca/data-access/. The raw microbiome data are publicly accessible under Accession: PRJNA685746. All requests will undergo an initial review by the GEM Project Operating Committee to ensure that the proposed use of samples or data aligns with the objectives of the original GEM Project Study Design and does not duplicate any ongoing analyzes within the nested cohort. If the proposal passes this stage, it will be forwarded to the GEM Project Steering Committee (GPSC) for broader scientific evaluation and discussion. This second-stage review will assess the overall scientific merit of the proposal, the availability of the requested samples or data, and the potential for overlap or conflict with current GEM Project aims. Applicants are encouraged to connect with a GPSC member who may be willing to support their application. Proposals that receive majority approval from the GPSC will be eligible to receive a letter of support from the GEM Project. This letter will indicate that the requested resources will be provided, contingent upon the applicant securing funding from a peer-reviewed granting agency. Review criteria include scientific significance, feasibility, and the availability of the necessary biological materials. Applicants should ensure their funding request includes costs related to sample processing and data access. The review process is designed to provide applicants with ample time to prepare for upcoming funding opportunities.
