# Supplementary material for: Fecal neuraminidase correlates with inflammatory signatures and microbiome shifts in preclinical Crohn’s disease
Source: Gut Microbes. 2026 Nov 5;18(1):2755166. doi: 10.1080/19490976.2026.2755166 (PMC13759132; doi:10.1080/19490976.2026.2755166)
Supplement: Supplementary file 1 [file KGMI_A_2755166_SM0856.docx]

**Supplementary Table 1:** Correlation of serum proteins with fecal neuraminidase activity

| **Protein** | **Correlation coefficient** | ***p* value** |
| --- | --- | --- |
| HO-1 | -0.33 | 0.0017 |
| GGT1 | -0.29 | 0.0047 |
| PRSS27 | -0.28 | 0.0082 |
| FABP2 | -0.27 | 0.0096 |
| F7 | -0.26 | 0.0112 |
| NOTCH3 | -0.24 | 0.0216 |
| CRTAC1 | -0.24 | 0.0247 |
| CNTN1 | -0.23 | 0.0271 |
| PON3 | -0.23 | 0.0274 |
| DNER | -0.22 | 0.0325 |
| TCN2 | -0.22 | 0.0334 |
| ANPEP | -0.22 | 0.0354 |
| MMP2 | -0.22 | 0.0404 |
| HSD11B1 | -0.21 | 0.0421 |
| CA5A | -0.21 | 0.0511 |
| PLCB1 | -0.19 | 0.0655 |
| LILRB5 | -0.19 | 0.0658 |
| SCGB3A2 | -0.19 | 0.0677 |
| SCF | -0.19 | 0.0687 |
| TRAP | -0.19 | 0.0695 |
| APOM | -0.19 | 0.0756 |
| CHL1 | -0.19 | 0.0787 |
| TNXB | -0.18 | 0.0806 |
| PTPRS | -0.18 | 0.0814 |
| CES1 | -0.18 | 0.0817 |
| CDSN | -0.18 | 0.0881 |
| MEPE | -0.18 | 0.0943 |
| IGFBP3 | -0.18 | 0.0948 |
| ITGA11 | -0.18 | 0.0951 |
| KIT | -0.17 | 0.0996 |
| CKAP4 | 0.17 | 0.1039 |
| TRIM21 | 0.18 | 0.0963 |
| LGALS9 | 0.18 | 0.0884 |
| DEFA1 | 0.19 | 0.0742 |
| MMP10 | 0.19 | 0.0712 |
| CTSL1 | 0.19 | 0.0684 |
| CDCP1 | 0.20 | 0.0534 |
| IL24 | 0.23 | 0.0291 |
| CXCL1.1 | 0.23 | 0.0268 |
| CXCL1 | 0.24 | 0.0221 |
| ACE2 | 0.25 | 0.0170 |
| IL1RL1 | 0.25 | 0.0155 |
| CCL5 | 0.25 | 0.0151 |
| CXCL11 | 0.25 | 0.0150 |

*p*-value was calculated using partial spearman correlation analysis. Only significant correlation defined as *p* value <0.1 are presented. None of the individual proteins remained statistically significant after false discovery rate (FDR) correction (all q > 0.05). Therefore, the nominally significant protein associations are presented as exploratory and hypothesis-generating.

**Abbreviation:** HO-1: Heme Oxygenase 1; GGT1: Gamma-Glutamyltransferase 1; PRSS27: Serine Protease 27; FABP2: Fatty Acid Binding Protein 2; F7: Coagulation Factor VII; NOTCH3: Notch Receptor 3; CRTAC1: Cartilage Acidic Protein 1; CNTN1: Contactin 1; PON3: Paraoxonase 3; DNER: Delta/Notch-Like EGF Repeat Containing; TCN2: Transcobalamin 2; ANPEP: Alanyl Aminopeptidase; MMP2: Matrix Metallopeptidase 2; HSD11B1: Hydroxysteroid 11-Beta Dehydrogenase 1; CA5A: Carbonic Anhydrase 5A; PLCB1: Phospholipase C Beta 1; LILRB5: Leukocyte Immunoglobulin Like Receptor B5; SCGB3A2: Secretoglobin Family 3A Member 2; SCF: Stem Cell Factor; TRAP: Tartrate-Resistant Acid Phosphatase; APOM: Apolipoprotein M; CHL1: Cell Adhesion Molecule L1 Like; TNXB: Tenascin XB; PTPRS: Protein Tyrosine Phosphatase Receptor Type S; CES1: Carboxylesterase 1; CDSN: Corneodesmosin; MEPE: Matrix Extracellular Phosphoglycoprotein; IGFBP3: Insulin Like Growth Factor Binding Protein 3; ITGA11: Integrin Subunit Alpha 11; KIT: KIT Proto-Oncogene Receptor Tyrosine Kinase; CKAP4: Cytoskeleton Associated Protein 4; TRIM21: Tripartite Motif Containing 21; LGALS9: Galectin 9; DEFA1: Defensin Alpha 1; MMP10: Matrix Metallopeptidase 10; CTSL1: Cathepsin L1; CDCP1: CUB Domain Containing Protein 1; IL24: Interleukin 24; CXCL1: C-X-C Motif Chemokine Ligand 1; ACE2: Angiotensin-Converting Enzyme 2; IL1RL1: Interleukin 1 Receptor Like 1; CCL5: C-C Motif Chemokine Ligand 5; CXCL11: C-X-C Motif Chemokine Ligand 11.

**Supplementary Table 2:** Correlation of bacterial taxa with fecal neuraminidase activity

| **Name** | **Coefficient** | **pvalue** | **qvalue** |
| --- | --- | --- | --- |
| Oscillospirales UCG.010 | -0.41 | 1.1443E-06 | 1.3274E-04 |
| Clostridia vadinBB60 group | -0.35 | 2.8582E-05 | 1.1052E-03 |
| Oscillospiraceae NK4A214 group | -0.36 | 1.9939E-05 | 1.1052E-03 |
| Eubacterium coprostanoligenes group | -0.34 | 4.4639E-05 | 1.2945E-03 |
| Butyricicoccaceae UCG.009 | -0.34 | 6.5457E-05 | 1.5186E-03 |
| Rikenellaceae Alistipes | -0.32 | 1.9830E-04 | 3.5996E-03 |
| Oscillospiraceae UCG.005 | -0.31 | 2.1722E-04 | 3.5996E-03 |
| Oscillospiraceae Flavonifractor | 0.31 | 3.1461E-04 | 4.5619E-03 |
| Clostridia UCG.014 | -0.29 | 7.1042E-04 | 9.1565E-03 |
| Ruminococcaceae Ruminococcus | -0.29 | 7.9534E-04 | 9.2260E-03 |
| Lachnospiraceae Ruminococcus gnavus group | 0.28 | 1.2747E-03 | 1.3281E-02 |
| Oscillospiraceae UCG.002 | -0.27 | 1.3739E-03 | 1.3281E-02 |
| Ruminococcaceae Angelakisella | -0.26 | 2.0242E-03 | 1.8062E-02 |
| Lachnospiraceae Lachnoclostridium | 0.24 | 5.2318E-03 | 4.3349E-02 |

Partial Spearman correlation analysis with adjustment for nested clustered group was performed Correlation coefficients and nominal *p* values were calculated for all feature pairs, and false discovery rate (FDR) adjustment was performed using the Benjamini-Hochberg method. Only significant correlation defined as *q* value <0.05 are presented.

**Supplementary Figure 1.** Comparison of fecal neuraminidase activity in stool samples from healthy matched control (HMC) and subjects who later developed pre-CD in subgroups. Subgroup analysis was conducted based on the median time to diagnosis duration (3.28 years), categorizing pre-CD participants into two groups: those diagnosed earlier (34 pre-CD) or later than the median duration (34 pre-CD) compared to their respective HMC. For conditional logistic regression analyses, fecal neuraminidase activity was standardized using Z-score transformation. Non-transformed values are shown in figures. The Y-axis represents the neuraminidase activity, expressed as mU/mg of protein in human stool samples. Bar height represents the median, with the error bar representing the interquartile range (IQR). The box spans from Q1 to Q3 and encompasses the median of the data. Each dots represent the fecal neuraminidase activity of a given participant. *P* values were calculated using a two-sided Wilcoxon rank-sum test (Mann-Whitney U).

**Abbreviation:** HMC: healthy matched control; CD: Crohn’s disease; IQR: interquartile range.

**Supplementary Figure 2.** Freezer storage time is not correlated with fecal neuraminidase activity. (a) Correlation between freezer storage time and fecal neuraminidase activity in individuals who subsequently developed Crohn’s disease (Pre-CD). (b) Correlation between freezer storage time and fecal neuraminidase activity in healthy matched controls (HMC). Spearman’s rank correlation coefficients (ρ) and corresponding *p* values are indicated. Each point represents an individual sample, with a line included to illustrate the trend.

**Abbreviation:** FNA: fecal neuraminidase activity; HMC: healthy matched controls; CD: Crohn’s disease.

**Supplementary Figure 3**. Correlation between fecal neuraminidase activity (FNA) and fecal calprotectin (FCP). FNA was positively correlated with FCP in individuals who later developed Crohn’s disease (Pre-CD) (ρ = 0.43, *p* = 0.00026) (a), but not in healthy matched controls (HMC) (ρ = 0.11, *p* = 0.36) (b). Each point represents an individual sample, with a line included to illustrate the trend. Rho (ρ) represents the correlation coefficient

**Abbreviation:** FNA: fecal neuraminidase activity; FCP: fecal calprotectin; CD: Crohn’s disease; HMC: healthy matched controls.

**Supplementary Figure 4**. Scatter plot showing the relationship between fecal neuraminidase activity in human stool samples (x-axis) and urinary fractional extraction of lactulose/mannitol ratio (LMR) (y-axis). Each point represents an individual sample, with a line included to illustrate the trend. Partial spearman correlation was performed to assess the correlation of fecal neuraminidase activity (FNA) with LMR (ρ = 0.12, *p*=0.17, n=127).

**Abbreviation:** FNA: fecal neuraminidase activity; LMR: lactulose/mannitol ratio
