# Supplementary material for: Fecal neuraminidase correlates with inflammatory signatures and microbiome shifts in preclinical Crohn’s disease
Source: Gut Microbes. 2026 Nov 5;18(1):2755166. doi: 10.1080/19490976.2026.2755166 (PMC13759132; doi:10.1080/19490976.2026.2755166)
Supplement: Supplementary file 2 [file KGMI_A_2755166_SM9261.docx]

**Supplementary methods**

**GEM study design**

The GEM Project is a large-scale, multi-center prospective research initiative aimed at understanding the complex interactions between genetic, environmental, and microbial factors in gastrointestinal disease development. By following healthy individuals, including first-degree relatives (FDR) of CD patients, and comparing them with those who later develop CD (defined as pre-CD), the GEM Project seeks to identify early biomarkers associated with disease onset.

This study focuses on a subset of the GEM cohort recruited between 2008 and 2020. More precisely the nested case-control cohort, is composed of each FDR who later developed CD by February 2020 (n=69) paired with a FDR who remained healthy during the same observation period (n=69). These pairs were matched based on age at recruitment, sex, geographic location (determined by postal and country codes), and duration of follow-up.

Prior to recruitment, all participants underwent screening to ensure the absence of gastrointestinal symptoms and any known diagnoses or indications of inflammatory bowel disease, celiac disease, or irritable bowel syndrome. The exclusion criteria for recruitment included: unintentional weight loss of over 15% of baseline weight in the past three months; any history of chronic or recurrent gastrointestinal or bowel disease; abdominal pain occurring more than once a week for over three months in the past year; diarrhea exceeding three times per day for more than three months in the last year; presence of blood in most stools; diagnosis of diabetes, celiac disease, irritable bowel syndrome, or inflammatory bowel disease; and significant symptoms indicative of gastrointestinal disease. Demographic details and environmental risk factors were collected, as detailed previously (refer to Supplementary Note 1-2). Written informed consent was obtained from all participants or their guardians prior to study enrollment. Ethical approval was obtained from the Mount Sinai Hospital Research Ethics Board (Toronto-Managing Center) and local recruitment centers (refer to the GEM Project Research Consortium section for a list of centers). Participants were contacted every six months via phone calls. In case a participant reported a diagnosis of CD, confirmation was sought from their treating physician based on clinical, endoscopic, radiographic, and/or histologic assessments (Supplementary Note 3).

**Fecal neuraminidase activity assessment**

Aliquots were prepared in an anaerobic chamber, and approximately 100 mg of fecal samples were homogenized in ~1.9 mL of pre-reduced phosphate-buffered saline (PBS) by thorough vortexing for 1 minute. The supernatant from the homogenized samples was collected to remove debris and then aliquoted into multiple cryovials. These aliquots were subsequently transferred to a −80 °C freezer for long-term storage. Fecal neuraminidase activity was measured using the Neuraminidase Assay Kit (Abcam, MA, USA; catalog number ab138888) according to the manufacturer’s instructions. The assay was performed by incubating the stool sample supernatants with the substrate provided in the kit. Briefly, the non-fluorescent neuraminidase substrate becomes fluorescent upon cleavage by endogenous neuraminidase. The fluorescence signal was measured using a microplate reader at an excitation/emission wavelength of approximately 320/450 nm (GloMax Multi Detection System, Promega, Madison, WI, USA), with a lower detection limit of 0.3 mU/mL neuraminidase. A standard curve was generated using the neuraminidase standard provided in the kit. The fecal neuraminidase activity level in each individual stool sample was measured in triplicate.

Protein concentration was determined using the Bradford assay. The Bradford reagent was added to the samples, and after a 10-minute incubation, the absorbance was measured at 595 nm (GloMax Multi Detection System, Promega, Madison, WI, USA). A standard curve was generated using known concentrations of bovine serum albumin (BSA). The protein level in each individual stool sample was measured in triplicate.

Fecal neuraminidase activity was then normalized to the protein concentration in each sample and expressed as mU per milligram of protein in human stool samples. This normalization corrects for variations in protein content, allowing for accurate comparisons of fecal neuraminidase activity across different samples. One pre-CD stool sample did not meet the detection limit of the protein assay and was therefore excluded from the analysis, resulting in a final sample size of 68 for the pre-CD group.

Sample selection and randomization were performed independently of the laboratory personnel conducting the enzymatic assays. Each matched Pre-CD and HMC pair was analyzed on the same assay plate to minimize inter-plate variability, while sample positions within each plate were randomized. Laboratory personnel performing the assays were blinded to sample identity and group allocation until completion of the assays and data analysis.

**Assessment of FCP**

Fecal calprotectin (FCP) concentrations were quantified using a commercially available enzyme-linked immunosorbent assay (ELISA) kit (BÜHLMANN fCAL, Schönbuch, Switzerland), following the manufacturer’s protocol. Approximately 50–100 mg of stool was homogenized in the provided extraction buffer and vortexed for 30 minutes to ensure adequate mixing. The homogenate was centrifuged at high speed for 5 minutes, and the resulting supernatant was carefully collected. A secondary centrifugation step of equal duration was performed to enhance sample clarity. The clarified supernatant was then subjected to ELISA, and absorbance was recorded at 450 nm using a calibrated microplate reader.

FCP concentrations were calculated based on duplicate readings, and the mean value was used for subsequent analysis. The dataset was winsorized according to manufacturer range of detection with values capped at1800 µg/g, and those below 30 µg/g were set to 30 µg/g.

**Serum protein quantification (OLINK platform)**

Proteomic profiling was conducted on baseline serum samples using the proximity extension assay (PEA) platform combined with quantitative real-time PCR (Olink® Proteomics, Uppsala, Sweden). This technology utilizes paired antibodies conjugated to unique DNA oligonucleotides that bind to their target proteins. When the antibody pairs are brought into close proximity upon binding to the same protein, a DNA polymerase-mediated extension reaction occurs, generating a unique DNA amplicon that is subsequently quantified by real-time PCR.

A total of 446 protein biomarkers were analyzed using five Olink® Target 96 panels, as referenced in the previous publication. Measurements below the limit of detection (LOD) for each protein were imputed using the Multivariate Imputation by Chained Equations (MICE) algorithm (v3.13.0) implemented in R, accounting for approximately 1.5% of the total 170,200 data points. Data points that failed Olink’s assay-specific quality control criteria were classified as missing, in accordance with the manufacturer’s guidelines. This approach enabled the quantification of 446 proteins across the cohort.

**Taxonomic profiling of the gut microbiota**

Fecal DNA was extracted using the QIAamp DNA Stool Mini Kit (Qiagen, Hilden, Germany) with slight modifications to the manufacturer’s protocol, as described in the previous literature. The V4 hypervariable region of the bacterial 16S rRNA gene was amplified using the 515F/806R primer pair and sequencing was performed in paired-end mode (2 × 150 base pairs) on the MiSeq platform (Illumina Inc, San Diego, CA). The resulting reads were demultiplexed and processed using the QIIME 2 (version 2.0) software and the DADA2 pipeline with default parameters. Taxonomic classification of the amplicon sequences was performed using the QIIME2 q2-feature-classifier plugin against the Silva 132 database of bacterial 16S rRNA sequences.

After quality filtering, 25,144 amplicon sequence variants (ASVs) were identified across all phylogenetic levels. Unassigned ASVs were removed, leaving 21,220 ASVs. Singleton ASVs was discarded, resulting in 17,629 ASVs. The sequences are available under PRJEB14839.

To address zero inflation in 16S rRNA microbiome data, we followed previously described strategies. Taxa with a prevalence of less than 20% across samples were excluded from further analysis. For features with baseline prevalence between 20% and 80%, the relative abundance data were transformed into binary variables, representing presence or absence in each sample. Features with a baseline prevalence above 80% were treated as continuous variables. For continuous variables, the odds ratio reflects the effect size per 0.01-unit increase in relative abundance, while for binary variables, the odds ratio represents the effect size of feature presence versus absence.

**Supplementary Figure 1.** Comparison of fecal neuraminidase activity in stool samples from healthy matched control (HMC) and subjects who later developed pre-CD in subgroups. Subgroup analysis was conducted based on the median time to diagnosis duration (3.28 years), categorizing pre-CD participants into two groups: those diagnosed earlier (34 pre-CD) or later than the median duration (34 pre-CD) compared to their respective HMC. For conditional logistic regression analyses, fecal neuraminidase activity was standardized using Z-score transformation. Non-transformed values are shown in figures. The Y-axis represents the neuraminidase activity, expressed as mU/mg of protein in human stool samples. Bar height represents the median, with the error bar representing the interquartile range (IQR). The box spans from Q1 to Q3 and encompasses the median of the data. Each dots represent the fecal neuraminidase activity of a given participant. *P* values were calculated using a two-sided Wilcoxon rank-sum test (Mann-Whitney U).

**Abbreviation:** HMC: healthy matched control; CD: Crohn’s disease; IQR: interquartile range.

**Supplementary Figure 2.** Freezer storage time is not correlated with fecal neuraminidase activity. (a) Correlation between freezer storage time and fecal neuraminidase activity in individuals who subsequently developed Crohn’s disease (Pre-CD). (b) Correlation between freezer storage time and fecal neuraminidase activity in healthy matched controls (HMC). Spearman’s rank correlation coefficients (ρ) and corresponding *p* values are indicated. Each point represents an individual sample, with a line included to illustrate the trend.

**Abbreviation:** HMC: healthy matched control; CD: Crohn’s disease.

**Supplementary Figure 3**. Correlation between fecal neuraminidase activity (FNA) and fecal calprotectin (FCP). FNA was positively correlated with FCP in individuals who later developed Crohn’s disease (Pre-CD) (ρ = 0.43, *p* = 0.00026) (a), but not in healthy matched controls (HMC) (ρ = 0.11, *p* = 0.36) (b). Each point represents an individual sample, with a line included to illustrate the trend. Rho (ρ) represents the correlation coefficient.

**Abbreviation:** FNA: fecal neuraminidase activity; FCP: fecal calprotectin; CD: Crohn’s disease; HMC: healthy matched control.

**Supplementary Figure 4**. Scatter plot showing the relationship between fecal neuraminidase activity in human stool samples (x-axis) and urinary fractional extraction of lactulose/mannitol ratio (LMR) (y-axis). Each point represents an individual sample, with a line included to illustrate the trend. Partial spearman correlation was performed to assess the correlation of fecal neuraminidase activity (FNA) with LMR (ρ = 0.12, *p*=0.17, n=127).

**Abbreviation:** FNA: fecal neuraminidase activity; LMR: lactulose/mannitol ratio

**Data Availability**

Requests for access to raw or analyzed data must comply with the guidelines outlined at <http://www.gemproject.ca/data-access/>. The raw microbiome data are publicly accessible under Accession: PRJNA685746. All requests will undergo an initial review by the GEM Project Operating Committee to ensure that the proposed use of samples or data aligns with the objectives of the original GEM Project Study Design and does not duplicate any ongoing analyses within the nested cohort. If the proposal passes this stage, it will be forwarded to the GEM Project Steering Committee (GPSC) for broader scientific evaluation and discussion.

This second-stage review will assess the overall scientific merit of the proposal, the availability of the requested samples or data, and the potential for overlap or conflict with current GEM Project aims. Applicants are encouraged to connect with a GPSC member who may be willing to support their application.

Proposals that receive majority approval from the GPSC will be eligible to receive a letter of support from the GEM Project. This letter will indicate that the requested resources will be provided, contingent upon the applicant securing funding from a peer-reviewed granting agency. Review criteria include scientific significance, feasibility, and the availability of the necessary biological materials. Applicants should ensure their funding request includes costs related to sample processing and data access. The review process is designed to provide applicants with ample time to prepare for upcoming funding opportunities.

**Credit Authorship Contributions**

Kaiwen Mu, PhD (Conceptualization: Lead; Formal analysis: Lead; Methodology: Lead; Visualization: Lead; Writing – original draft: Lead; Writing – review & editing: Lead).

Cong Phi Dang, PhD (Methodology: supporting; Writing – review & editing: supporting).

Mingyue Xue, PhD (Methodology: supporting; Writing – review & editing: supporting).

Hien Q. Huynh, MD (Conceptualization: Equal; Recruitment: Equal; Writing – review & editing: supporting).

Anne M. Griffiths, MD (Conceptualization: Equal; Recruitment: Equal; Writing – review & editing: supporting).

Dan Turner, MD, PhD (Conceptualization: Equal; Recruitment: Equal; Writing – review & editing: supporting).

Paul Moayyedi, MD, PhD (Conceptualization: Equal; Recruitment: Equal; Writing – review & editing: supporting).

A. Hillary Steinhart, MD (Conceptualization: Equal; Recruitment: Equal; Writing – review & editing: supporting).

Levinus A. Dieleman, MD, PhD (Conceptualization: Equal; Recruitment: Equal; Writing – review & editing: supporting).

Colette Deslandres, MD (Conceptualization: Equal; Recruitment: Equal; Writing – review & editing: supporting).

Alain Bitton, MD (Conceptualization: Equal; Recruitment: Equal; Writing – review & editing: supporting).

David R. Mack, MD (Conceptualization: Equal; Recruitment: Equal; Writing – review & editing: supporting).

Kevan Jacobson, MBBCh (Conceptualization: Equal; Recruitment: Equal; Writing – review & editing: supporting).

Maria Cino, MD (Conceptualization: Equal; Recruitment: Equal; Writing – review & editing: supporting).

Guy Aumais, MD (Conceptualization: Equal; Recruitment: Equal; Writing – review & editing: supporting).

Remo Panaccione, MD (Conceptualization: Equal; Recruitment: Equal; Writing – review & editing: supporting).

Brian Bressler, MD (Conceptualization: Equal; Recruitment: Equal; Writing – review & editing: supporting).

Sun-Ho Lee, MD, PhD (Formal analysis: Supporting; Methodology: Supporting; Writing – review & editing: supporting).

Williams Turpin, PhD (Funding acquisition: Lead; Conceptualization: Lead; Formal analysis: Supporting; Methodology: Supporting; Project administration: Lead; Supervision: Lead; Writing – original draft: Lead; Writing – review & editing: Lead).

Ken Croitoru, MD (Funding acquisition: Lead; Conceptualization: Lead; Formal analysis: Supporting; Methodology: Supporting; Project administration: Lead; Resources: Lead; Supervision: Lead; Writing – original draft: Lead; Writing – review & editing: Lead).

**Acknowledgments:** The authors thank the members of the Crohn’s and Colitis Canada Genetic Environmental Microbial Project Global Project Office. The CCC GEM Project Research Consortium includes: Maria Abreu,^1^ Paul Beck,^2^ Charles Bernstein,^3^ Kenneth Croitoru,^4,5^ Levinus A. Dieleman,^6^ Brian Feagan,^7^ Anne Griffiths,^8^ David Guttman,^9^ Kevan Jacobson,^10^ Gilaad Kaplan,^2^ Denis O. Krause,^11^ Karen Madsen,^12^ John Marshall,^13^ Paul Moayyedi,^13^ Mark Ropeleski,^14^ Ernest Seidman,^15^ Mark Silverberg,^4^ Scott Snapper,^16^ Andy Stadnyk,^17^ Hillary Steinhart,^4^ Michael Surette,^18^ Dan Turner,^19^ Thomas Walters,^20^ Bruce Vallance,^21^ Guy Aumais,^22^ Alain Bitton,^15^ Maria Cino,^4^ Jeff Critch,^23^ Lee Denson,^24^ Colette Deslandres,^25^ Wael El-Matary,^26^ Hans Herfarth,^27^ Peter Higgins,^28^ Hien Huynh,^29^ Jeffrey S. Hyams,^30^ David Mack,^31^ Jerry McGrath,^32^ Anthony Otley,^33^ and Remo Panancionne^34^; from the ^1^ Division of Gastroenterology, Department of Medicine, University of Miami, Miller School of Medicine, Miami, Florida; ^2^ Department of Medicine, University of Calgary, Calgary, Alberta, Canada; ^3^ Inflammatory Bowel Disease Clinical and Research Centre and Department of Internal Medicine, Max Rady College of Medicine, Rady Faculty of Health Sciences, University of Manitoba, Manitoba, Winnipeg, Canada; ^4^ Division of Gastroenterology & Hepatology, Temerty Faculty of Medicine, University of Toronto, Toronto, Ontario, Canada; ^5^ Zane Cohen Centre for Digestive Diseases, Mount Sinai Hospital, Toronto, Ontario, Canada; ^6^ Division of Gastroenterology, Department of Medicine, University of Alberta, Edmonton, Alberta, Canada; ^7^ Departments of Medicine, Epidemiology, and Biostatistics, University of Western Ontario, London, Ontario, Canada; ^8^ Division of Gastroenterology, The Hospital for Sick Children, Toronto, Ontario, Canada; ^9^ Centre for the Analysis of Genome Evolution and Function, University of Toronto, Toronto, Ontario, Canada; ^10^Research Institute, British Columbia Children’s Hospital, Vancouver, British Columbia, Canada; ^11^Department of Medical Microbiology and Infectious Diseases, University of Manitoba, Winnipeg, Manitoba, Canada; ^12^Division of Gastroenterology, Department of Medicine, Faculty of Medicine and Dentistry, University of Alberta, Edmonton, Alberta, Canada; ^13^Department of Medicine, Farncombe Family Digestive Health Research Institute, McMaster University, Hamilton, Ontario, Canada; ^14^Gastrointestinal Diseases Research Unit, Department of Medicine, Queen’s University, Kingston, Ontario, Canada; ^15^Division of Gastroenterology and Hepatology, McGill University Health Centre, Montreal, Quebec, Canada; ^16^Division of Gastroenterology, Hepatology and Nutrition, Boston Children’s Hospital and Harvard Medical School, Boston, Massachusetts; ^17^Department of Microbiology and Immunology, Dalhousie University, Halifax, Canada; ^18^Department of Biochemistry and Biomedical Sciences, McMaster University, Hamilton, Ontario, Canada; ^19^The Juliet Keidan Institute of Pediatric Gastroenterology and Nutrition, Shaare Zedek Medical Center, The Hebrew University of Jerusalem, Jerusalem, Israel; ^20^Division of Gastroenterology, Hepatology and Nutrition, The Hospital for Sick Children, Toronto, Ontario, Canada; ^21^BC Children’s Hospital Research Institute, University of British Columbia, Vancouver, British Columbia, Canada; ^22^Biostatistics Department, Princess Margaret Cancer Centre, University Health Network, Toronto, Ontario, Canada; ^23^Janeway Children’s Health and Rehabilitation Centre and Department of Pediatrics, Memorial University, St. John’s, Newfoundland, Canada; ^24^Department of Pediatrics, Cincinnati Children’s Hospital Medical Center, Cincinnati, Ohio; ^25^Centre Hospitalier Universitaire Sainte-Justine, Montreal, Quebec, Canada; ^26^Pediatric Gastroenterology, Max Rady College of Medicine, University of Manitoba, Manitoba, Winnipeg, Canada; ^27^Division of Gastroenterology and Hepatology, University of North Carolina at Chapel Hill, Chapel Hill, North Carolina; ^28^Division of Gastroenterology & Hepatology, University of Michigan, Ann Arbor, Michigan; ^29^Department of Pediatrics, University of Alberta, Edmonton, Alberta, Canada; ^30^Division of Digestive Diseases, Hepatology, and Nutrition, Connecticut Children’s Medical Center, Hartford, Connecticut; ^31^Division of Gastroenterology, Hepatology & Nutrition, Children’s Hospital of Eastern Ontario and University of Ottawa, Ottawa, Ontario, Canada; University of British Columbia, Vancouver, British Columbia, Canada; ^32^Provincial Medical Genetics Program, Eastern Health, St. John’s, Newfoundland, Canada; ^33^Division of Gastroenterology, Izaak Walton Killam Hospital, Dalhousie University, Halifax, Nova Scotia, Canada; and ^34^Inflammatory Bowel Disease Unit, University of Calgary, Calgary, Alberta, Canada

We acknowledge the use of the Niagara supercomputer at the SciNet HPC Consortium, funded by the Canada Foundation for Innovation, the Government of Ontario, Ontario Research Fund - Research Excellence, and the University of Toronto.
